# The importance of adherence in tuberculosis treatment clinical trials and its relevance in explanatory and pragmatic trials

**DOI:** 10.1371/journal.pmed.1002884

**Published:** 2019-12-10

**Authors:** Andrew Vernon, Katherine Fielding, Rada Savic, Lori Dodd, Payam Nahid

**Affiliations:** 1 Clinical Research Branch, Division of TB Elimination, NCHHSTP, US Centers for Disease Control & Prevention, Atlanta, Georgia, United States of America; 2 TB Centre, London School of Hygiene & Tropical Medicine, London, United Kingdom; 3 Faculty of Health Sciences, University of Witwatersrand, Johannesburg, South Africa; 4 Department of Bioengineering and Therapeutic Sciences, University of California, San Francisco, California, United States of America; 5 National Institute for Allergy and Infectious Disease, National Institutes of Health, Washington DC, United States of America; 6 Division of Pulmonary and Critical Care Medicine, University of California, San Francisco at San Francisco General Hospital, San Francisco, California, United States of America

## Abstract

Andrew Vernon and co-authors discuss adherence to therapy and its measurement in tuberculosis treatment trials.

**Summary Points**

- Adherence to prescribed treatment remains a critical component of clinical trials in tuberculosis (TB) treatment. Recent evidence indicates that adherence strongly influences the outcome of therapy; attention to its quantification and measures to assure its implementation should increase.
- In the context of a World Health Organization (WHO) Technical Consultation on “Advances in Clinical Trial Design for Development of New TB Treatments,” we reviewed the challenges related to adherence confronting the trials community.
- We discuss the importance of adherence to therapy in TB clinical trials, consider several definitions and measures of adherence, comment on the standard provided by directly observed therapy (DOT), and briefly review evolving electronic methods for the assessment of adherence.
- Adherence affects both the outcome of therapy and the risk of acquired drug resistance. Assessment of adherence should consider not only overall adherence but also the timing and intensity of nonadherence.
- Appropriate methods for pooling and analyzing electronic data on adherence are needed.
- Better methods are needed for linking information on adherence to individual pharmacokinetics and pharmacodynamics and to individual patient outcomes.

## Introduction

Medication adherence remains the most underrated and understudied factor affecting the outcome of tuberculosis (TB) therapy. Its importance has been appreciated since the time of the initial South India trial conducted by the Tuberculosis Research Center and the British Medical Research Council (MRC), comparing in-patient and domiciliary treatment [1]. Twenty-five years later, Fox wrote “It is paradoxical to insist on the importance of 100% success with primary chemotherapy and to use self-administered chemotherapy as a means of achieving it” [2]. In their 1999 encyclopedic review of the MRC TB trials, Fox, Mitchison, and Ellard reported that a common feature of those trials was “the effort made [including hospitalization for the full treatment] to ensure that the patients actually took the prescribed regimen throughout the trial period” [3]. These examples illustrate the importance of adherence to treatment for the validity of a clinical trial and for the success of individual and programmatic care. Despite the clear and obvious need to ensure optimal treatment adherence, "full supervision," in the form of directly observed therapy (DOT) as currently delivered, has not consistently been associated with improved outcomes. Thus, significant challenges persist in measuring and maximizing adherence with antituberculosis therapy; recent data and analyses provide evidence that the absence of full adherence in TB trials has important implications for TB regimen development and for the durability of new regimens. In March 2018, the World Health Organization (WHO) held a Technical Consultation on Advances in Clinical Trial Design for Development of New TB Regimens, which is the topic of the Collection of which this paper is part [4]. In this context, we reviewed the importance of treatment adherence, the implications of a drug or regimen’s “forgiveness for missed doses,” and emerging novel approaches to measuring and maximizing adherence in clinical trials and in patient care.

## Importance of adherence

Adherence affects patient outcomes and is thus an important factor to consider when evaluating regimens in clinical trials. Differing adherence across treatment arms could potentially lead to misleading conclusions about treatment arm performance. For example, consider (as a hypothetical example) a study with poor adherence in the control arm but perfect adherence in the experimental arm. If the goal of a study is to measure the efficacy of a new regimen, the relatively poor adherence in the control arm will give an overly optimistic estimate of the improvement in outcomes with the experimental treatment. However, if the goal of the study is to evaluate effectiveness (i.e., performance under real-word conditions), the relative difference in adherence may accurately reflect the real-word difference in the 2 regimens. One complication is that the level of adherence may vary widely across different populations and cultural or economic settings, raising concerns about whether estimates of effectiveness are broadly generalizable. The relation of adherence to regimen effectiveness (the usual target outcome of “pragmatic” trials) in trial versus program settings was noted nearly 50 years ago and continues to challenge the generalizability of trial findings [5].

Adherence may have a substantial impact on the interpretation of clinical trial findings. Adherence is often an active choice by each patient on how to comply with the assigned therapy. Adjustments in analysis based on observed adherence may alter the balance introduced by randomization. Restricting analyses only to those with high adherence focuses on a subset of the population that may have fundamentally different risk than those who are not adherent. A classic example of this circumstance is provided by the Coronary Drug Project trial assessing a lipid-lowering drug in men with recent myocardial infarction: participants with good adherence had low and equivalent mortality in the test and placebo arms, whereas poor adherers did better in the test arm [6]. Still, understanding trial outcomes among participants who take drugs as prescribed (i.e., a “per protocol [PP] analysis”) has some appeal, even though such an analysis is not protected by randomization. In recent TB treatment trials, adequate adherence was defined by a threshold of 76%–80% of intended doses taken, to identify the PP population [7,8]. This is consistent with analytic practice in the reporting of the MRC trials (which defined an “excessive interruption” with exclusion from the relapse analyses if less than approximately 77% of intended doses were received) [9] and with recent practice in prominent United States TB control programs (e.g., New York City) [10].

A recent meta-analysis of three Phase III trials of fluoroquinolone-based 4-month TB treatment regimens found that nonadherence was the single most potent factor associated with unfavorable treatment outcome. The adjusted hazard ratios (aHRs) were 5.7 (95% CI 3.3–9.9) for test arm participants who missed 10% or more of prescribed doses and 1.4 (95% CI 1.0–1.9) for test arm participants who had less than 10% nonadherence, compared with participants who completed treatment without any missed doses; the aHRs were similar in the control arm participants (Table 1) [11]. The same trend was seen in PP analysis, which excluded participants who failed to complete at least 75%–80% of intended doses.

**Table 1:** Pooled mITT analysis of 3 TB treatment-shortening trials showing impact of adherence on unfavorable outcome.

	Test arms (4 months, with FQ)		Control arms (6 months, no FQ)	
Prescribed doses	Unfavorable	Total	Unfavorable	Total
**Received 100% of prescribed doses**	238 (18%)	1,348	85 (9%)	913
**Received 90%–99% of prescribed doses**	64 (22%)	288	37 (16%)	230
**Received <90% of prescribed doses**	15 (47%)	32	16 (37%)	43

Abbreviations: FQ, fluoroquinolones; mITT, modified intent-to-treat

Such a potent influence of nonadherence serves to emphasize the often-noted importance of the quality of performance in noninferiority trials; it further suggests that PP analyses might examine more than 1 threshold for nonadherence (e.g., 80% and 95%) to help in more robustly assessing efficacy. A stronger analytic approach might evaluate the effect on trial outcomes of baseline pre-randomization variables associated with poor adherence [12]; by definition, baseline variables should be approximately balanced in large randomized trials, thereby not introducing bias in the assessment of outcomes.

## Definitions and measures of adherence

Adherence refers to the completeness with which participants or patients follow medical instructions. Because adherence can vary so greatly among different individuals, it can have an important influence on treatment outcomes. Adherence more broadly may also involve changes required by the protocol (e.g., in response to elevated liver function tests) that are not active choices by the participant. In their recent review on this topic, Blaschke and colleagues observe that adherence is a major source of variability affecting the outcome of TB therapy [13]. Adherence, in turn, is affected by diverse individual and social factors [14]. Other sources of variability include the formulation of the test medications, the prescribed dosing, and the pharmacokinetics and pharmacodynamics of each agent employed, as well as key features of the infecting *Mycobacterium tuberculosis* strains (for example, the minimal inhibitory concentrations of each drug employed), and inherent characteristics of the host patient (including genetic determinants of drug metabolism, immunologic competence, and the architecture of TB lesions). The latter sources of variability are already determined at the onset of therapy and are therefore likely to be balanced between treatment arms by the process of randomization. In contrast, adherence is subject to ongoing variability during treatment, which complicates its effects. Although genetic factors affecting drug exposure should be comparable at randomization, their impact may vary by the drugs used in each arm. The recent availability of electronic methods for monitoring adherence has made it possible to measure adherence quite precisely; these novel methods have become the gold standard for compiling dosing histories. At least 3 aspects of adherence are specifically relevant to antituberculosis therapy [15]:

1. the total quantity of nonadherence (i.e., what proportion of doses are missed, in relation to the total number of doses in the intended treatment regimen?);
2. the timing of nonadherence (i.e., does it occur at the outset of therapy, throughout therapy, or primarily at the end of the intended course of therapy?); and
3. the intensity and patterns of nonadherence (i.e., are many consecutive doses missed, or are missed doses distributed relatively evenly throughout the course of therapy?).

The third aspect in particular can exert an important influence upon drug pharmacokinetics and thus may predispose to either loss of efficacy or emergence of drug resistance. Consecutive lapses in dosing can lead to lower-than-usual peak drug concentrations and lower total drug exposures, whereas extra doses can result in risk of toxicity due to higher-than-usual peak concentrations and total exposures (the review by Blaschke and colleagues includes a figure that nicely illustrates these risks [13]). The term “forgiveness” of a regimen is intended to reflect the impact of variable lapses in dosing. Although “forgiveness” was originally defined as “the post dose duration of therapeutically effective drug action, minus the recommended interval between doses” [13], the shift from action to no action is likely to be gradual and to vary among patients.

In the circumstance of treatment for TB, several examples come readily to mind:

1. The work of Imperial and colleagues demonstrated the association of overall nonadherence with the treatment outcome of short-duration fluoroquinolone-based regimens [11];
2. The timing of nonadherence is likely critical, because nonadherence in the presence of high bacillary loads typically seen in the intensive phase is likely to have greater impact than the same degree of nonadherence later during the continuation phase, when bacillary loads are generally several logs lower; this is particularly an issue in the presence of immunosuppression, because bacillary multiplication will resume more rapidly when such patients become nonadherent;
3. Similarly, a gap of several doses (i.e., intensity) would likely have greater impact in the presence of high bacillary loads, such as during the early intensive phase. Recent guidelines have advised against the use of highly intermittent regimens for this reason, with substantial supporting evidence [16].

There is thus an urgent need for improved measures and more sophisticated means of analyzing such patterns and types of nonadherence in relation to treatment outcomes. In Phase I and Phase II studies, optimal adherence is imperative to make decisions on regimens to move forward to late-stage development. In Phase III studies, the objectives would drive the decision on adherence implementation and measurement. In both scenarios, there is a need to measure and report adherence appropriately, to understand better the performance of the tested interventions.

## Methods for assessment of adherence

Currently available methods for assessing adherence are limited, but considerable work is underway to develop better approaches (Table 2). Among methods that have been used in past investigations are (1) clinic-based DOT, in which ingestion of each medication dose is observed in clinic by a health worker, thereby allowing exact counting of each study dose given or missed; (2) home-based DOT by a health worker, a community worker, or a family member; (3) adherence to the calendar of study visits; (4) patient self-reports of adherence; (5) use of pill counts made at the time of study visits; (6) electronic bottle caps or similar methods to quantify the number and time of opening of medication bottles; and (7) tests of blood, urine, or other body materials for specific drugs or their metabolites.

**Table 2:** Strengths and weaknesses of methods for encouraging and/or assessing adherence.

Methods	Description	Strengths	Weaknesses
**Home-based or work-based DOT by HCW**	Ingestion of each medication dose is observed and recorded by an HCW at home/work.	Monitors adherence in real time; convenience for patient.	Cost^c^; patient confidentiality.
**In clinic DOT by HCW**	Ingestion of each medication dose is observed and recorded by an HCW at the clinic.	Monitors adherence in real time; lower cost than home or work-based DOT by HCW.	Inconvenience to patient; cost to health system.
**Family member DOT**	Ingestion of each medication dose is observed and recorded by a designated family member.	Convenience for patient; lower cost versus DOT by health worker.	Confidence in the reports from family members; data on real-time adherence not available unless transmitted to HCW on a daily basis.
**Live video DOT [27,28]**	Ingestion of each medication dose is videoed by patient and observed by an HCW in real time^a^.	Monitors adherence in real time; convenience for patient.	Cost (HCW review of live video; smartphone); patient and HCW acceptability.
**Recorded video DOT**	Ingestion of each medication dose is videoed by patient and sent to HCW to be viewed later^a^.	Convenience for patient.	Cost (HCW review; smartphone); patient and HCW acceptability; depending on when videos are viewed, may not monitor adherence in real time; privacy concerns.
**Direct monitoring:** **1. Blood testing**	Blood sample taken to measure plasma levels of TB medications.	Direct measurement of dose ingested.	Feasibility/logistics; cost; depends on timing of blood sample relative to time of ingestion; limited time window.
**2. Urine testing**	Urine testing for drug metabolites (e.g., isoniazid).	Direct measurement of dose ingested.	Feasibility/logistics; cost; sensitivity may vary depending on acetylator status; limited time window.
**3. Swallowed pill sensors [29]**	Ingestible sensor embedded in TB medications. Pill interacts with gastric acid, and a signal is transmitted to adhesive monitor on patient, which in turn transmits information to smartphone.	No reliance on sample collection.	Cost; relies on patient wearing the adhesive monitor; patient acceptability.
**Indirect monitoring, device facilitated** **Pill box [22]** **Bottle cap [30]**	Medications placed in pill box/bottle. Opening/closing of box/bottle, a proxy for dose taken, is documented in real time via SIM card.^b^	Monitors adherence in real time (if pill box/bottle cap transmits); low cost (relative to HCW DOT).	Pill box opening/bottle cap removal may not reflect an ingestion of dose; nonopening may not reflect noningestion of dose if medications are not stored in box/bottle.
**Indirect monitoring, patient facilitated** **SMS text messages**	Patient sends SMS message to HCW when a dose has been ingested.	Monitors adherence in real time; low cost.	Patient needs to be familiar with text messaging; text message sent may not reflect an ingestion of dose; nonreceipt of SMS may not reflect noningestion of dose.

^a^ HCW observation could be replaced by face recognition and motion-detection software.

^b^ Non–real time use of pillbox also possible where data on pill box opening/closing are downloaded at regular intervals, at a pharmacy refill, for example.

^c^ Costs are important for all modalities; these often vary by setting or country and vary for newer technologies.

Abbreviations: DOT, directly observed therapy; HCW, healthcare worker; SIM, subscriber identity module; SMS, short message service; TB, tuberculosis

Some methods offer direct demonstration of adherence, whereas others provide only indirect readouts. Newer methods are currently under investigation, including the quantification of drug levels in hair [17] and the measurement of changes in skin color associated with specific medications (e.g., rifamycins or clofazimine); these both indicate cumulative adherence rather than dynamic patterns.

There were several early trials of interventions to improve adherence (with the goal of achieving better outcomes), but the knowledge base overall remains sparse, and recent systematic reviews underline the need for further investigation and a substantially enlarged evidence base [18]. Electronic methods for measuring or estimating adherence are increasingly available, and some TB programs and countries are moving forward rapidly with such digital tools, generating large quantities of data. Appropriate methods for pooling and analyzing these data are needed, as are methods for linking information on adherence to individual pharmacokinetics and pharmacodynamics and to individual outcomes [19]. A recently published systematic review of newer digital technologies, including short message service (SMS), video-observed therapy (VOT), and medication monitors (MMs), for TB treatment adherence identified few comparative studies for inclusion and concluded that the evidence on the effect of digital technologies to improve TB care remained limited [20]. For the studies included in the review, no statistically significant effect on treatment completion was identified when SMS was added to standard care or when VOT was used as an alternative to in-person DOT. It was noted that MMs increased the probability of cure (risk ratio 2.3, 95% CI 1.6–3.4) in one observational study [21] and in one trial, significantly reduced missed treatment doses relative to standard care (adjusted means ratio 0.58, 95% CI 0.42–0.79) [22].

Overall, the systematic review concluded that more studies of better quality are needed for the evaluation of technologies applicable to measuring and maximizing adherence. There are also few studies that have assessed the accuracy of digital adherence technologies in measuring ingestion of medication doses. A study in China assessed the MM box (box opening between 6 and 24 hours before urine sample taken) against detecting rifampicin in urine and found a sensitivity of 99% and specificity of 95% [23]. In India, SMS responses (from the cellphone-based monitoring system known as “99DOTS”) over a 48-hour period, indicating dose taken, were compared with isoniazid detection in urine, and a sensitivity and specificity of 68% and 62% were observed, respectively [24]. A recent randomized trial in the United Kingdom noted considerable success in use of VOT to assure dosing, compared with traditional DOT [25]. Adequate study designs for evaluating accuracy of adherence monitoring devices are critical to provide realistic tests of performance. Bias may be introduced, for example, if patient knowledge that a urine sample will be collected inflates adherence around the scheduled time. Unannounced collections may mitigate this. Timing of collections throughout the full treatment period may also be important, for example, if adherence drops later during treatment. The recent technical consultation report on “Advances in Clinical Trial Designs for Development of New TB Treatments” also strongly endorsed the need for further investigations in this domain and noted that trials offer an excellent platform for substudies in these areas [26].

Evidence for the benefit of traditional DOT has not been entirely consistent, and its role remains controversial [18,31,32]. Some investigators favor relatively strict application of in-person DOT, whereas others feel this is excessive and does not contribute to achievement of objectives in properly randomized and implemented trials. Some investigators favor implementation of non-family-member in-person DOT, whereas others feel it is more reasonable to allow local determination of what types of adherence support would be most useful. Better means to measure adherence and its association with outcomes would contribute usefully to this discussion [15,16]. Some of the digital health approaches being assessed in pragmatic trials may be combined with differentiated care; in this approach, for example, those identified as poor adherers through the digital health measures are assisted further with more traditional approaches to maximizing adherence, with actual observation by health workers in the most extreme cases [25,33].

Likewise, there is no consensus on a single criterion for “clinically important” nonadherence. Assessment of the degree of nonadherence that should be deemed “clinically important” depends on multiple factors specific to each trial setting, including the component drugs of the regimen, the dosing schedule, the pharmacokinetics of the individual drugs, and other risk factors and comorbidities that could influence the risk of treatment failure or relapse. Embedded in this discussion is consideration of the concept of “forgiveness” of a regimen (i.e., as noted previously, a reference to the types and levels of nonadherence that would not substantively alter the likelihood of treatment effectiveness of a regimen). Although it could be considered that this aspect should be reflected in the regimen’s efficacy and requires no other adjustment, it can be argued that this aspect should be considered in the determination of the noninferiority margin [34]. Further, some note that in the rational design and composition of new TB regimens, the “forgiveness” of a regimen for missing doses should be considered with significantly greater deliberation than is currently common, particularly given that adherence in practice will never be perfect.

## Adherence and acquired drug resistance

Recently released WHO target regimen profiles (TRPs) for TB identify the barrier to emergence of resistance as an important characteristic to address in the development of new drugs and regimens [35]. The association of nonadherence with acquisition of drug resistance has been well reviewed [36,37,38], but the mechanisms underlying the association remain largely speculative [39]. In the WHO TRPs, it is suggested, based on expert opinion, that each component of the regimen should permit no greater mutation rate (in unselected bacterial populations) than 1/10^7^ mutations/bacterium/generation and that new resistance to one or more drugs in the regimen should emerge in fewer than 2% of treatment courses when taken as prescribed and when there is no preexisting resistance to the drugs in the regimen. This minimal target is based on an acquired resistance rate of 0%–2% when 5 effective drugs are used in the WHO-recommended multiply drug resistant (MDR) regimen [40]. The reality of reduced adherence in the field, as compared with clinical trial settings, and the potential impact such real-world usage of a regimen may have on risks for emergence of resistance need further study, representing another outcome of interest in how much “forgiveness” a putative new regimen may carry for missed doses.

## Summary

In conclusion, medication adherence remains a critical, yet understudied, factor influencing outcomes of TB therapy. Its importance has been recognized since the advent of effective antituberculosis therapy, and the vital role adherence plays in the conduct of TB clinical trials has been further highlighted in contemporary clinical trials [11]. The growing importance of noninferiority trial designs and the challenge of interpreting PP analyses have focused more attention on the issue of precisely measuring adherence and adherence patterns. Adherence is important in both superiority and noninferiority trials and in both intent-to-treat (ITT) and per-protocol (PP) analyses; both should be performed, and both should assess the impact of variation in adherence. Only recently has our ability to measure adherence improved. Novel (in particular, electronic) methods for assessing and encouraging adherence hold promise, and efforts to develop a robust evidence base to support them are growing. Our understanding of the impact of nonadherence on key outcomes (treatment success, emergence of resistance) in TB treatment trials is relatively modest but is also receiving increased attention from investigators. Continued development of more convenient, more reliable, and less costly means to achieve high levels of adherence will serve both trials and programs well.

## Abbreviations

aHR: adjusted hazard ratio
DOT: directly observed therapy
HCW: healthcare worker
ITT: intent-to-treat
MDR: multiply drug resistant
mITT: modified ITT
MM: medication monitor
MRC: British Medical Research Council
PP: per protocol
SMS: short message service
TB: tuberculosis
TRP: target regimen profile
VOT: video-observed therapy
WHO: World Health Organization
